# Area of residual tumor (ART) can predict prognosis after post neoadjuvant therapy resection for pancreatic ductal adenocarcinoma

**DOI:** 10.1038/s41598-019-53801-2

**Published:** 2019-11-20

**Authors:** Satoshi Okubo, Motohiro Kojima, Yoko Matsuda, Masayoshi Hioki, Yasuhiro Shimizu, Hirochika Toyama, Soichiro Morinaga, Naoto Gotohda, Katsuhiko Uesaka, Genichiro Ishii, Mari Mino-Kenudson, Shinichiro Takahashi

**Affiliations:** 1 Division of pathology, Research Center for Innovative Oncology, National Cancer Center Hospital East, Chiba, Japan; 2 Department of Hepatobiliary and Pancreatic surgery, National Cancer Center Hospital East, Chiba, Japan; 3 Department of Pathology, Tokyo Metropolitan Geriatric Hospital and Institute of Gerontology, Sakae-cho, Itabashi-ku, Tokyo, Japan; 4 Department of Gastroenterological Surgery, Fukuyama City Hospital, Okayama, Japan; 5 Department of Gastroenterological Surgery, Aichi Cancer Center Hospital, Aichi, Japan; 6 Division of Hepato-Biliary-Pancreatic Surgery, Department of Surgery, Kobe University Graduate School of Medicine, Hyogo, Japan; 7 Department of Gastrointestinal Surgery, Kanagawa Cancer Center, Kanagawa, Japan; 8 Division of Hepato-Biliary-Pancreatic Surgery, Shizuoka Cancer Center, Shizuoka, Japan; 9 Department of Pathology, Massachusetts General Hospital and Harvard Medical School, Boston, MA USA

**Keywords:** Surgical oncology, Prognostic markers

## Abstract

An increasing number of patients with pancreatic ductal adenocarcinoma (PDAC) have undergone resection after neoadjuvant therapy (NAT). We have reported Area of Residual Tumor (ART) as a useful pathological assessment method to predict patient outcomes after post NAT resection in various cancer types. The aim of this study was to assess the prognostic performance of ART in PDAC resected after NAT. Sixty-three patients with PDAC after post NAT resection were analyzed. The viable residual tumor area was outlined and the measurement of ART was performed using morphometric software. The results were compared with those of the College of American Pathologist (CAP) regression grading. Of 63 cases, 39 (62%) patients received chemoradiation therapy and 24 (38%) received chemotherapy only. The median value of ART was 163 mm^2^. Large ART with 220 mm^2^ as the cut-off was significantly associated with lymphatic invasion, vascular invasion and perineural invasion, while CAP regression grading was not associated with any clinicopathological features. By multivariate analysis, large ART (≥220 mm^2^) was an independent predictor of shorter relapse free survival. Together with our previous reports, an ART-based pathological assessment may become a useful method to predict patient outcomes after post NAT resection across various cancer types.

## Introduction

Pancreatic ductal adenocarcinoma (PDAC) is a lethal disease, and the clinical outcome is the worst among gastrointestinal cancers in the world^1^. Currently, curative surgical resection is the only chance for prolonged survival, but only less than 20% of patients present at a resectable stage^2,3^. Furthermore, even when potentially curative resections are achieved, the 5-year survival rate after resection is only 8% to 25% due to high recurrence rates^3–7^. Neoadjuvant therapy (NAT) has been associated with down-staging and margin-negative resections leading to potential prognostic advantages in PDAC^8^. Furthermore, NAT will likely increase a rate of completion of multimodality therapy, achieve eradication of microscopic distant metastasis, and consequently improve cost-effectiveness^9^. Therefore, an increasing number of PDACs have been surgically resected after NAT in practice, and several phase II clinical trials of neoadjuvant chemoradiotherapy and chemotherapy have been conducted for borderline resectable and locally advanced PDACs, and even for resectable tumors^10–16^. Currently, NAT for PDAC is considered as a part of standard care in many institutions and will likely contribute to improving patient outcomes in the future.

Several imaging studies and serum tumor markers has been failed to predict residual tumor volumes after NAC due to treatment-related alterations of tumor microenvironment^17,18^. On the other hand, pathological assessments can identify residual tumor cells and morphological changes secondary to treatment; thus, they may also provide key outcome parameters in PDAC cases after NAT^19^. In fact, pathological assessments on tumor regression or residual tumor in resections after NAT have been proven to be useful in predicting patient outcomes in many cancer types including rectal, lung, and esophageal cancers^20–22^. In PDAC, several pathological assessment methods including the College of American Pathologist (CAP) regression grading and Evans grading system have been proposed^23–25^. However, studies on the clinical utility of these grading systems are still limited and there have been controversial results as to whether they could predict patient outcomes after post NAT resections^26–28^. Thus, it is important to establish a standard pathological assessment method that will contribute to the prediction of clinical outcomes and ultimately to the management of PDAC patients.

We have reported area of residual tumor (ART) as a novel objective and quantitative pathological assessment method to evaluate the residual tumor in resections after NAT for gastric, lung and rectal cancers^29–31^. In addition, a practical semi-quantitative assessment method as a surrogate of ART has also been proposed for rectal cancer^30^. However, no study has evaluated a role of ART in predicting outcomes of patients with PDAC after post NAT resection. The aim of this study was to assess the prognostic value of ART in comparison to CAP regression grading that is currently considered as a standard pathological assessment for residual tumor in post NAT resections for PDAC.

## Results

### Patient demographics

The study cohort consisted of 38 men and 25 women, with a median age of 65 years (range, 38–78 years) (Table 1). All 63 patients underwent surgical resection with curative intent after NAT. Of 63 cases, the diagnosis of resectable, borderline resectable, locally advanced and metastatic disease before NAT were 12 (19%), 34 (54%), 13 (21%), 4 (6%), respectively. Thirty-nine (62%) patients received preoperative chemoradiation therapy and 24 (38%) received preoperative chemotherapy only. Chemotherapeutic agents used for preoperative chemoradiation were S-1 in 38 (60%) patients and gemcitabine in 1 (2%), and those for preoperative chemotherapy were gemcitabine + S-1 in 12 (19%) patients, gemcitabine + nab-paclitaxel in 6 (10%), other gemcitabine-based regimens in 4 (6%) and S-1 only in 2 (3%).

**Table 1:** Characteristics of post neoadjuvant resections for PDAC patients.

Characteristics	Total
	n = 63 (100%)
Age (y),	
median (range)	65 (38–78)
≥70	21 (34%)
Sex (male)	38 (60%)
Tumor location	
head/body and tail	47 (75%)/16 (24%)
Preoperative diagnosis	
R/BR/LA/M	12 (19%)/34 (54%)/13 (21%)/4 (6%)
Preoperative treatment	
CRT/CT	39 (62%)/24 (38%)
Foamy gland alteration	
<10%/≥10%	55 (87%)/8 (13%)
Mucus lake	
<10%/≥10%	55 (87%)/8 (13%)
Fibrosis	
<25%/≥25%	25 (40%)/38(60%)
Foamy macrophage	
Positive/Negative	9 (14%)/54 (86%)
Cholesterol cleft	
Positive/Negative	4 (6%)/59 (94%)
Calcification	
Positive/Negative	1(2%)/62 (98%)
Tumor differentiation	
G1/G2/G3/GX	20 (32%)/34 (54%)/8 (13%)/1 (2%)
Lymphatic invasion	
Negative/Positive	31 (49%)/32 (51%)
Vascular invasion	
Negative/Positive	22 (35%)/41 (65%)
Perineural invasion	
Negative/Positive	14 (22%)/49 (78%)
Stage	
0/IA/IB/IIA/	1 (2%)/20 (32%)/15 (24%)/0 (0%)/
IIB/III/IV	19 (30%)/7(11%)/1(2%)
Resection margin negative	54 (86%)
CAP regression grade	
0 or 1/2 or 3	9 (14%)/54 (86%)
Area of residual tumor (mm^2^)	
median (range)	161 (0–526)

PDAC: Pancreatic Ductal Adenocarcinoma; R: Resectable; BR: Borderline Resectable; LA: Locally advanced; M: Metastasis; CRT: Chemoradiation therapy; CT: Chemotherapy; CAP: College of American Pathologists.

### Histological factors

The median value of ART was 161 mm^2^ (range, 0–526) (Table 1). the only one patient had complete pathologic response with no residual tumor (ART: 0). Correlation between ART and preoperative tumor size by CT was fair (Spearmann’s rank correlation coefficient r = 0.42, P = 0.003) in NCCHE cohort. The cut-off value of ART was determined as 220 mm^2^ by ROC curve (Area under the curve = 0.70, sensitivity: 0.45 and Specificity: 0.81). Large ART (>220 mm^2^) was found in 23 (37%) patients and small ART (≤220 mm^2^) was in 40 (63%) patients. Tumor regression in accordance with the CAP regression grading was grade 0 or 1 in 9 (14%) patients and grade 2 or 3 in 54 (86%) patients. As for the pathological features that have been reported in association with therapeutic effects, foamy gland changes present in more than 10% of residual tumor cells were seen in 8 (13%) patients, mucus lake occupying more than 10% of the tumor tissue in 8 (13%) patients, and fibrosis replacing more than 25% of the tumor area in 38 (60%) patients. Foamy macrophages, cholesterol clefts and calcifications were seen in 14%, 6% and 2% of the study cohort, respectively. Large ART was significantly associated with the presence of lymphatic invasion, vascular invasion and perineural invasion and advanced TNM stage, while the CAP regression grading showed no correlation with these clinicopathologic factors (Table 2). None of the features that have been reported as treatment effects (foamy gland alteration, mucus lake, fibrosis, foamy macrophages, cholesterol clefts and calcifications) was associated with ART or the CAP regression grading.

**Table 2:** Characteristics of post neoadjuvant resections for PDAC patients classified by ART value and CAP regression grade.

Characteristics	ART ≤ 220 mm^2^	ART > 220 mm^2^	P value	CAP: 0, 1	CAP: 2, 3	P value
	n = 40 (100%)	n = 23 (100%)		n = 9 (100%)	n = 54 (100%)	
Age (y),			0.71			0.36
median (range)	65 (38–78)	66 (51–78)		65 (38–74)	65 (40–78)	
≥70	14 (35%)	7 (30%)		2 (22%)	19 (35%)	
Sex			0.64			0.08
male	25 (63%)	13 (57%)		3 (33%)	35 (65%)	
Tumor location			0.11			0.64
head	28 (70%)	20 (87%)		7 (78%)	41 (76%)	
Preoperative diagnosis			0.19			0.54
R or BR	27 (68%)	19 (83%)		7 (78%)	39 (72%)	
Preoperative treatment			0.34			0.53
CRT	23 (58%)	16 (70%)		6 (67%)	33 (61%)	
Foamy gland alteration			0.62			0.27
≥10%	5 (13%)	3 (13%)		0 (0%)	8 (15%)	
Mucus lake			0.38			0.32
≥10%	6 (15%)	2 (9%)		2 (22%)	6 (11%)	
Fibrosis			0.03			0.22
≥25%	20 (50%)	18 (78%)		7 (78%)	31 (57%)	
Foamy macrophage			0.43			0.23
Positive	5 (13%)	4 (17%)		0 (0%)	9 (17%)	
Cholesterol cleft			0.54			0.07
Positive	3 (8%)	1 (4%)		0 (0%)	4 (7%)	
Calcification			0.64			0.86
Positive	1 (3%)	0 (0%)		0 (0%)	1 (2%)	
Tumor differentiation			0.38			0.08
G3	6 (15%)	2 (9%)		3 (33%)	5 (9%)	
Lymphatic invasion			<0.01			0.52
Positive	13 (33%)	18 (78%)		3 (33%)	27 (50%)	
Vascular invasion			<0.01			0.38
Positive	19 (48%)	22 (96%)		5 (56%)	36 (67%)	
Perineural invasion			<0.01			0.10
Positive	26 (65%)	23 (100%)		5 (56%)	44 (81%)	
Stage			<0.01			0.13
≥IB	20 (50%)	22 (96%)		4 (44%)	38 (70%)	
Resection margin			0.43			0.62
Negative	35 (88%)	19 (81%)		8 (89%)	46 (85%)	

PDAC: Pancreatic Ductal Adenocarcinoma; ART: Area of Residual Tumor; CAP: College of American Pathologists; R: Resectable; BR: Borderline Resectable; CRT: Chemoradiation therapy.

### Overall survival analysis

**Figure 1:**
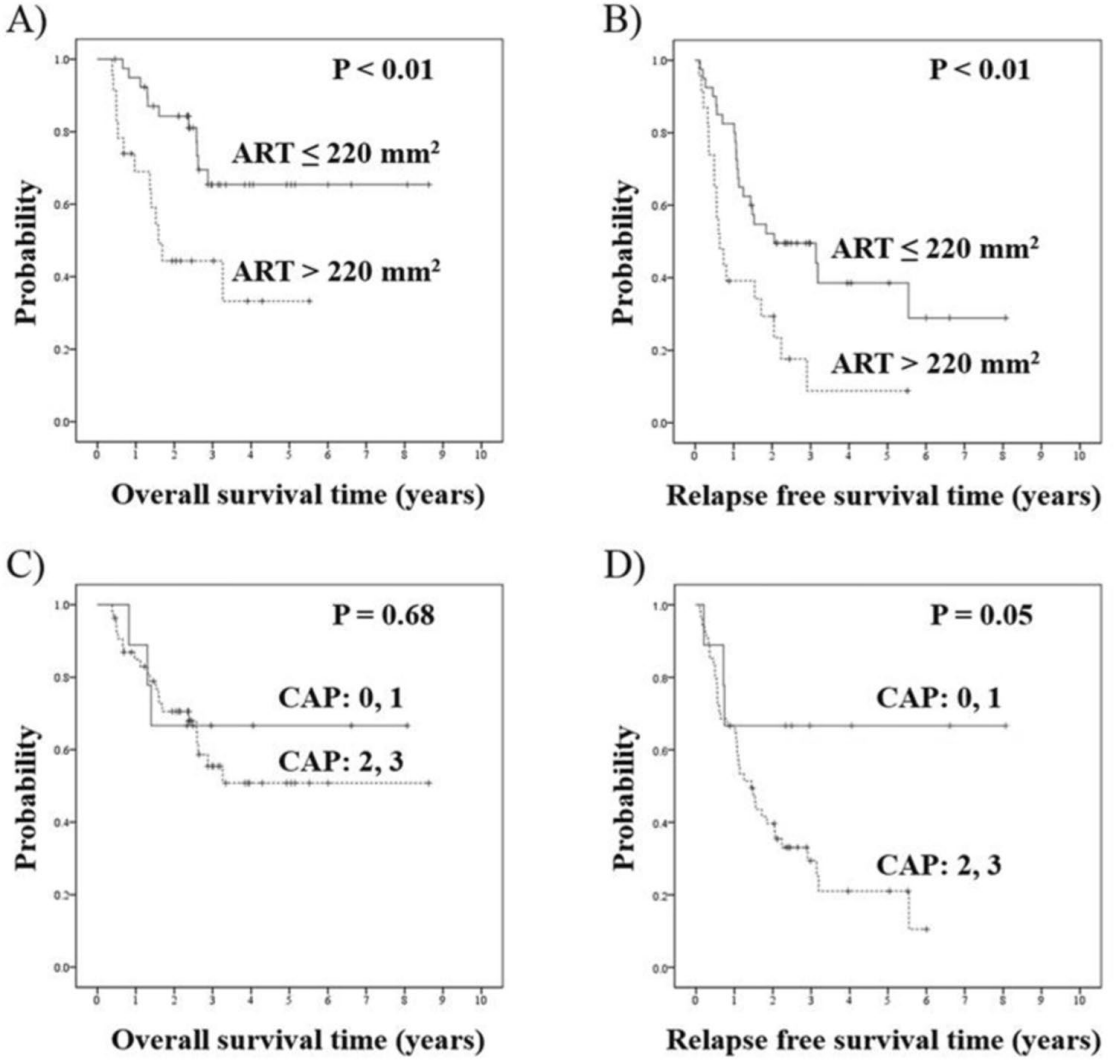
Survival curves of post neoadjuvant resections for PDAC patients. Overall survival time classified by ART value (**A**), by CAP regression grade (**C**), Relapse-free survival time classified by ART value (**B**), by CAP regression grade (**D**). PDAC: pancreatic ductal adenocarcinoma; CAP: College of American Pathologists; ART: Area of Residual Tumor.

For all 63 patients, 1-, 2-, and 3-year overall survival rates were 85%, 70%, and 57%, respectively. The median OS time were not reached for patients with small ART and 1.59 years for those with large ART. The 2-year OS rate was 84% for patients with small ART and 44% for those with large ART. Large ART was significantly associated with shorter OS compared to small ART by log-rank analysis, while the CAP regression grading had no bearing on OS (Fig. 1).

On univariate analysis, the predictors of shorter OS were vascular invasion, positive resection margin and large ART. However, no variable remained significant upon multivariate analysis (Table 3).

**Table 3:** Analyses of overall survival in post neoadjuvant resections for PDAC patients.

Characteristics		n	MST (Years)	Overall survival			
				Univariate		Multivariate	
				P-value	P-value	HR	(95%CI)
Sex	Male	38	3.26	0.61	0.79		
	Female	25	NR				
Age (y)	<70	42	NR	0.74	0.94		
	≥70	21	NR				
Tumor location	Head	48	NR	0.77			
	Body and tail	15	NR				
Preoperative diagnosis	R/BR	46	NR	0.61			
	LA/M	17	NR				
Preoperative treatment	CRT	39	NR	0.57			
	CT	24	NR				
Foamy gland alteration	<10%	55	NR	0.50			
	≥10%	8	2.59				
Mucus lake	<10%	55	NR	0.53			
	≥10%	8	NR				
Fibrosis	<25%	25	NR	0.56			
	≥25%	38	NR				
Foamy macrophage	Positive	9	NR	0.66			
	Negative	54	NR				
Cholesterol cleft	Positive	4	1.60	0.76			
	Negative	59	NR				
Calcification	Positive	1	NR	0.43			
	Negative	62	NR				
Tumor differentiation	G3	8	NR	0.61			
	G1/G2/GX	55	NR				
Lymphatic invasion	Positive	31	NR	0.47			
	Negative	32	NR				
Vascular invasion	Positive	41	2.60	0.01	0.15		
	Negative	22	NR				
Perineural invasion	Positive	49	NR	0.25			
	Negative	14	NR				
Stage	0 - IA	21	NR	0.75			
	IB - IV	42	NR				
Resection margin	Positive	9	1.52	0.05	0.07		
	Negative	54	NR				
CAP regression grade	0, 1	9	NR	0.68			
	2, 3	54	NR				
Area of residual tumor (mm^2^)	>220	23	1.59	<0.01	0.10		
	≤220	40	NR				

PDAC: Pancreatic Ductal Adenocarcinoma; MST: Median Survival Time; R: Resectable; BR: Borderline Resectable; LA: Locally advanced; M: Metastatic; CRT: Chemoradiation therapy; CT: Chemotherapy; CAP: College of American Pathologists; NR: Not Reached.

### Relapse-free survival analysis

1-, 2-, and 3-year RFS rates were 67%, 44%, and 35%, respectively. The median RFS time was 1.53 years for all 63 patients, 0.64 years for patients with large ART, and 2.06 years for those with small ART. RFS was significantly shorter for patients with large ART than for those with small ART (P < 0.01) (Fig. 1).

On univariate analysis, large ART, and CAP regression grade 2 or 3 were associated with shorter RFS, but on multivariate analysis, only large ART remained as an independent predictor of shorter RFS (Table 4).

**Table 4:** Analyses of relapse-free survival in post neoadjuvant resections for PDAC patients.

Characteristics		n	MRFS (Years)	Relapse-free survival			
				Univariate		Multivariate	
				P-value	P-value	HR	(95%CI)
Sex	Male	38	1.07	0.26	0.10		
	Female	25	2.05				
Age (y)	<70	42	1.53	0.98	0.74		
	≥70	21	1.49				
Tumor location	Head	48	1.53	0.84			
	Body and tail	15	1.11				
Preoperative diagnosis	R/BR	46	1.53	0.72			
	LA/M	17	1.49				
Preoperative treatment	CRT	39	1.53	0.23			
	CT	24	1.49				
Foamy gland alteration	<10%	55	1.53	0.54			
	≥10%	8	1.09				
Mucus lake	<10%	55	1.49	0.20			
	≥10%	8	NR				
Fibrosis	<25%	25	1.44	0.55			
	≥25%	38	1.55				
Foamy macrophage	Positive	9	1.06	0.60			
	Negative	54	1.53				
Cholesterol cleft	Positive	4	0.55	0.31			
	Negative	59	1.55				
Calcification	Positive	1	1.11	0.55			
	Negative	62	1.53				
Tumor differentiation	G3	8	0.71	0.72			
	G1/G2/GX	35	1.55				
Lymphatic invasion	Positive	31	1.44	0.51			
	Negative	32	1.53				
Vascular invasion	Positive	41	1.09	0.09			
	Negative	22	3.14				
Perineural invasion	Positive	49	1.44	0.17			
	Negative	14	3.19				
Stage	0 - IA	21	2.06	0.24			
	IB - IV	42	1.49				
Resection margin	Positive	9	0.81	0.22			
	Negative	54	1.72				
CAP regression grade	0, 1	9	NR	0.05	0.16		
	2, 3	54	1.44				
Area of residual tumor (mm^2^)	>220	23	0.64	<0.01	<0.01	2.77	1.46–5.25
	≤220	40	2.06				

PDAC: Pancreatic Ductal Adenocarcinoma; MRFS: Median Relapse-Free Survival; R: Resectable; BR: Borderline Resectable; LA: Locally advanced; M: Metastastatic; CRT: Chemoradiation therapy; CT: Chemotherapy; CAP: College of American Pathologists; NR: Not Reached.

## Discussion

The ideal pathologic assessment method for post NAT resections needs to be: 1) prognostic; 2) objective; 3) reproducible; 4) practical; 5) applicable across various cancer types.

To date, multiple grading systems including the CAP regression grading, Evans grading, and MD Anderson grading have been proposed to assess therapeutic effects in post NAT resections for PDAC^23,25,26^. These pathological regression grading systems have been reported to be useful in predicting patient outcomes after resection in some studies, while Lee *et al*. reported that the CAP grading system was not associated with prognosis in 167 patients with potentially resectable PDAC who had undergone post NAT resection^27^. Williams, *et al*. also reported that the CAP grading system was not associated with prognosis in 93 patients with locally advanced PDAC that had been resected after NAT^32^. Heinrich, *et al*. used the Evans grading system and reported that there was no difference in survival stratified by treatment effects among 25 patients with resectable PDAC^28^. Chuong, *et al*. evaluated 36 patients with borderline resectable PDAC using the MD Anderson grading and CAP grading systems and reported that the CAP grading system was not associated with prognosis, but the MD Anderson grading predicted OS and RFS^33^. However, only univariate analysis was performed in their study and the study cohort was relatively small. In the current study, the CAP regression grading was not associated with either patient outcomes or any clinicopathologic factors.

It is important to note that the original tumor area and biology before treatment need to be estimated in the currently available pathological assessment methods for post NAT resections. For instance, the Evans grading system assesses destroyed tumor cells secondary to the treatment, while differentiation of treatment effects from programed death of tumor cells that are not associated with the treatment may be challenging. Similarly, the CAP regression grading system evaluates tumor regression compared to the (estimated) original tumor area. In this context, tumor bed characterized by fibrosis is often used as a surrogate marker for tumor area before treatment^34^. Generally, it is expected that effective treatments would induce tumor cell death resulting in fibrosis; however, desmoplasia in the tumor tissue present before the therapy may also remain after the therapy^35^. In addition, there are many other sources of fibrosis associated with PDAC including pre-existing chronic pancreatitis or secondary chronic pancreatitis due to obstruction of the pancreatic duct by tumor^36^. Furthermore, we have previously reported that therapeutic regimens influenced on the extent of fibrosis in rectal cancer, although fibrosis was not associated with patient outcomes^30^. In this study, we confirmed no association between the extent of fibrosis and patient outcomes after post NAT resections for PDAC, while Chun, *et al*. reported a proportion of fibrosis in the residual tumor was associated with prognosis^37^. The difference in the results between those studies may also indicate that the evaluation of fibrosis could be subjective; thus, its utility in estimating the tumor area before NAT and in predicting patient outcomes is controversial.

The commonly used assessment methods which estimate the tumor area and biology before therapy are also subject to interobserver variability among pathologists^19^. Concordance studies on various grading systems between pathologists revealed kappa-values to be 0.28–0.38 for the 3-tierd regression grading of rectal cancer and 0.18–0.40 for the CAP regression grading of PDAC^35,38^. We believe that these fair agreements among pathologists were associated in part with subjectivity in estimating the tumor area before therapy. Therefore, in this study, we tried to establish ART that minimizes any estimation in regression as a new regression assessment method for PDAC, and reported here that ART was useful in predicting patient outcomes after post NAT resection for PDAC. Large ART was associated with shorter RFS as well as aggressive pathologic features and advanced TNM stage. ART may play an important role in identifying patients who may have benefits from adjuvant therapy after post NAT resections.

**Figure 2:**
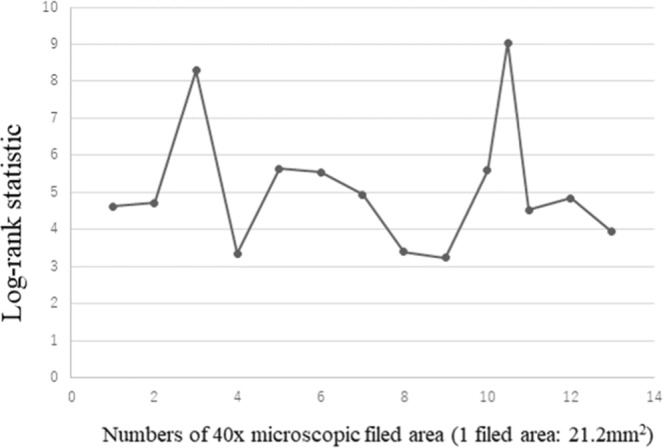
The log-rank statistics of various cut-offs equivalent to numbers of 40x microscopic filed area. Partitions greater than a 3.8 log-rank statistic correspond to a P value < 0.05. The partitions at 10.5 40x fields (nearly equal to 220mm^2^) generated the largest log-rank statistics, which have smallest P values (P < 0.01), and 3 40x fields (63.6 mm^2^) is the second highest log-rank statistics.

In this study, we used morphometric software to make the assessment of residual tumor as objective as possible. The morphometric analysis, however, may not be practical for a routine use; thus, a semi-quantitative ART-based assessment in accordance with the results of this study has been proposed^3^. In the semi-quantitative system, tumor regression is scored based on a number of microscopic fields replaced by residual tumor cells. After confirming that the surface area equivalent to a 40x field is 21.2 mm^2^ with several microscopes used in this study (BX50 Olympus, Japan), we evaluated the log-rank statistics of various cut-offs equivalent to numbers of 40x microscopic filed area (Fig. 2). The partitions at 10.5 40x fields (nearly equal to 220mm^2^) generated the largest log-rank statistics, which have smallest P values (P < 0.01), and 3 40x fields (63.6 mm^2^) is the second highest log-rank statistics. ART > 64 mm^2^ equivalent to 3 40x fields (63.6 mm^2^) is also significantly associated with shorter RFS in this study cohort (Fig. S1). Considering the practicality, we have planned to validate the utility of the ART-based regression grading system using microscopic fields (3 40x fields as a cut-off) in another cohort.

It is also important to note that ART may be useful across multiple cancer types. Currently, various pathological assessment methods have been used in individual organs. Therefore, it is very difficult to compare treatment effects of one treatment protocol across multiple organs. We have previously reported that ART assessment of therapeutic effects is useful in various organs including gastric, lung and rectal cancers and we also confirmed the utility of ART in PDAC in this study. Therefore, we believe that the ART-based grading system will contribute to the assessment on therapeutic effects of a treatment regimen applied to multiple tumor types meaning that it has a potential to become a standard assessment method for post NAC resections in general. It is important, however, to determine an appropriate and unified cut-off value of ART to make it applicable for multiple organs since our previous and current studies identified and used a wide range of cut-off values (from 50 mm^2^ to 400 mm^2^) for rectal, pancreas and lung cancers.

There are several limitations in this study. First of all, the number of cases used for analysis was relatively small. In addition, there were multiple regimens for NAT used in our cohort and given the small number of cases treated with each regimen, we couldn’t compare the difference in effects between the NAT regimens. Thus, we have planned to validate the predictive value of ART and evaluate the difference in patient outcomes between various treatment regimens using a larger cohort.

In conclusion, ART is a useful pathological assessment method to predict patient outcomes after post NAT resection for PDAC. Compared with the few grading systems that are currently available, ART is more objective and is applicable across various cancer types. Further, a more practical, semi-quantitative ART-based assessment measuring a number of microscopic fields replaced by residual tumor cells can be developed and may become a standard method for the evaluation of post NAT resection specimens in general in the future.

## Methods

### Informed consent

All experiments were performed after obtaining written comprehensive informed consents from all patients. This study was approved by the National Cancer Ethical Review Board (No. 2017–358), and was performed in accordance with relevant guidelines and regulations.

### Patients

We originally included 51 consecutive patients with PDAC who had undergone surgical resection after NAT from 2006 to 2016 at National Cancer Center Hospital East (NCCHE cohort) and 17 patients with PDAC who had taken part in the JASPAC05 trial (curative resection after NAT) at 5 institutions except NCCHE (JASPAC05 cohort) (13). After exclusion of 5 patients due to: 1) treatment-related death (n = 3); 2) concomitant malignancies (n = 1) and 3) unavailability of histologic slides (n = 1), 63 patients formed the study cohort. Clinicopathological data were collected retrospectively from patient medical records in the NCCHE cohort and from the data center in the JASPAC05 cohort. The present study was approved by the institutional review board of National Cancer Center (2017–358). In the NCCHE cohort, the median interval from the last treatment day to the operation day was 31 days (range; 13–145 days) and in the JASPAC05 cohort, all surgeries were performed within 15–56 days from the last treatment day. The median follow-up period was 3.0 years (95% confidence interval, 2.8–3.9 years). In the NCCHE cohort, indication of neoadjuvant therapy and operation was decided by a multidisciplinary discussion at tumor board. For resectable PDAC, upfront surgery was usually performed; however, the patient was treated with neoadjuvant therapy upon participation in a clinical trial of neoadjuvant therapy. For borderline resectable PDAC, preoperative chemoradiation or chemotherapy was first performed. After the neoadjuvant treatment, surgery with curative intent was performed if there was no metastatic disease depicted by CT and/or MRI. For locally advanced PDAC, patients were treated with chemotherapy, but operation was considered when the treatment effects had led to amelioration of vascular involvement, and tumor marker decreased to within normal limit. For metastatic PDAC, the patients underwent resection of the pancreatic primary with curative intent only when the chemotherapy had led to complete response of metastatic deposits depicted by CT or MRI.

### Histologic assessment

**Figure 3:**
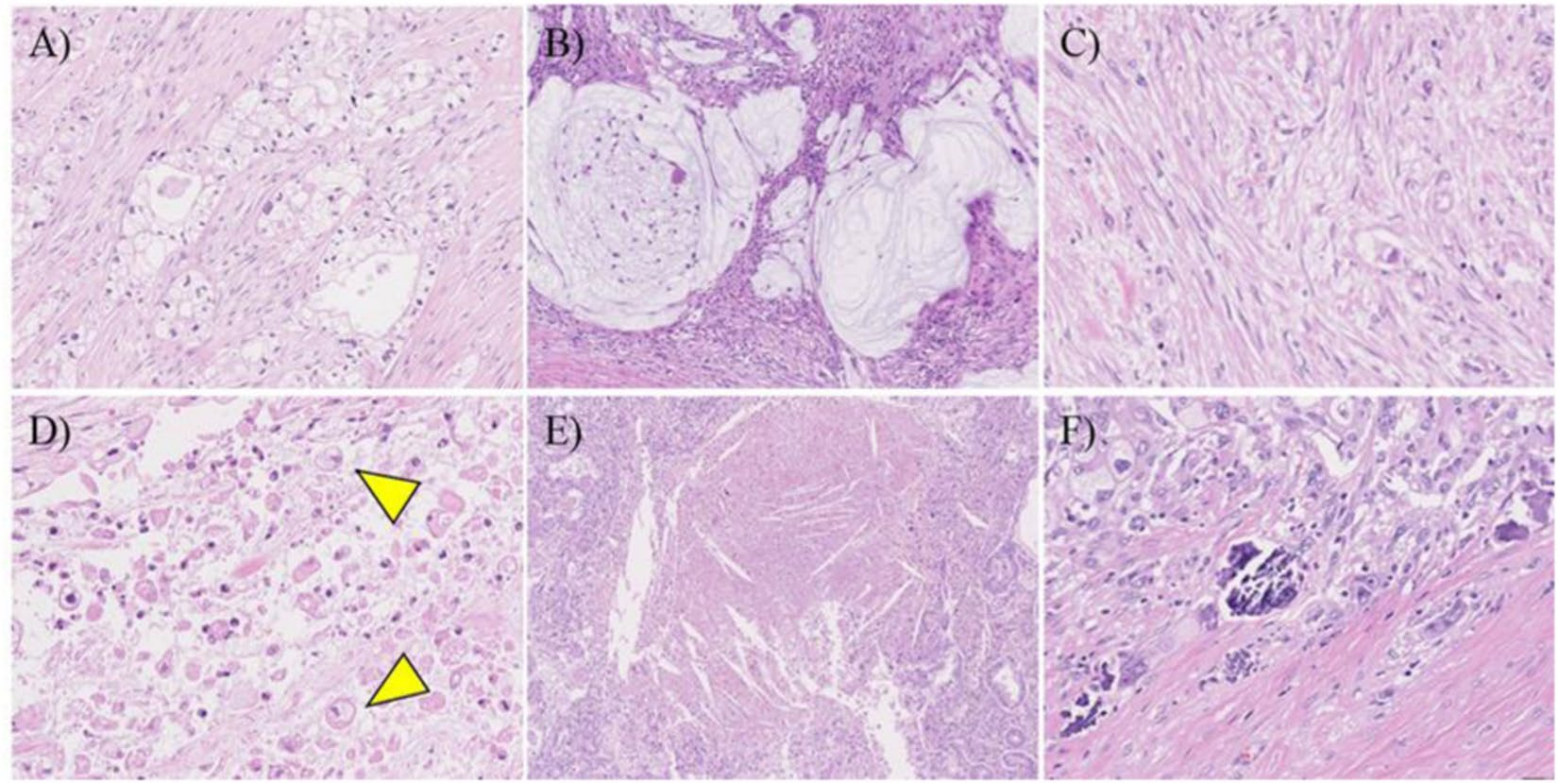
Pathological features associated with tumor regression (**A**) foamy gland pattern, (**B**) mucus lake (mucin pool), (**C**) fibrosis, (**D**) foamy macrophage (arrow head), (**E**) Cholesterol clefts, (**F**) calcification.

All tumor tissue was sliced with 4–7-mm intervals at all institutions, and all slices with tumor were entirely submitted for microscopic examination. Histological examination was performed using hematoxylin and eosin (H.E) staining and evaluated by two independent reviewers (S.O. and M.K.) who were blinded to clinical data. Discrepancies in evaluations between reviewers were resolved by discussion. Previously reported pathological features associated with therapeutic effects including foamy gland changes, mucus lake (mucin pool), fibrosis, foamy macrophages, cholesterol clefts and calcifications were assessed using all tumor slides (Fig. 3)^24,36,39,40^. Given that it is challenging to distinguish between treatment-related fibrosis and desmoplasia, any sources of fibrosis are assessed as fibrosis. The proportion of tumor cells with foamy gland changes, mucus lake, or any fibrosis was assessed in each case, and the cohort was divided into two groups using the cut-off level of 10% for foamy gland changes, 10% for mucous lake, and 25% for fibrosis, respectively^30^. Macrophages with foamy cytoplasm, cholesterol clefts, and calcifications were considered present when they were detected at x2 – x10 objective lens. The CAP grading system was also assessed as follows: grade 0, no viable cancer cells; grade 1, single cells or rare small groups of cancer cells; grade 2, residual tumor with evident tumor regression; grade 3, extensive residual tumor with no evident tumor regression^25^. In addition, all tumor slides were examined for the presence of lymphatic, vascular, and/or perineural invasion and margin status. Positive resection margin was defined as tumor cells present at the margin. Tumor-node-metastasis (TNM) classification was assessed according to the criteria outlined in the 8^th^ edition of the Union for International Cancer Control (UICC)^41^.

### Measurement of ART

**Figure 4:**
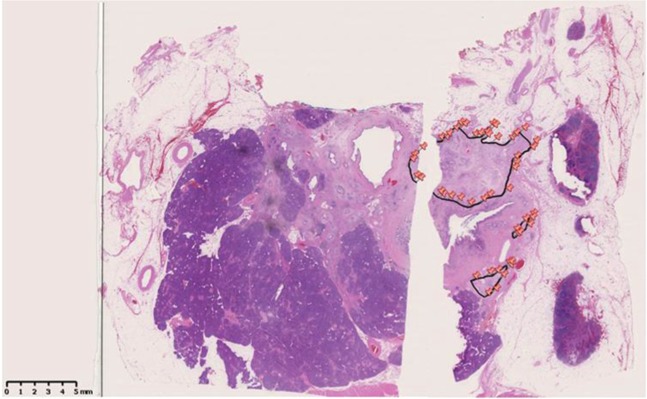
Representative example of measurement of ART. The viable residual tumor area was outlined and the measurement of ART was performed using morphometric software. ART: Area of Residual Tumor.

The measurement of ART was performed as follows: 1) All H&E slides from the largest slice with residual tumor that was determined during the histologic assessment were digitally scanned in each case. 2) A viable residual tumor area was outlined and its surface area was calculated using a NanoZoomer Digital Pathology Virtual Slide Viewer (Hamamatsu Photonics, Hamamatsu, Japan, scanned by x40 ocular lens). Necrotic tumor cells and fibrosis was not included in the measurement. *In situ* lesions and acellular mucous lake was also excluded from the measurement in this study. 3) Isolated, viable tumor foci more than >2 mm apart from the largest tumor area in the slide were also identified and measured individually. The sum of the tumor areas was defined as ART. 4) The cut-off value of ART was determined using ROC curve (Fig. 4).

### Definition of clinical outcomes

Overall survival (OS) was calculated from the date of surgery to that of death from any cause. Relapse-free survival (RFS) was defined as the period from the date of surgery to that of tumor relapse or death of any cause, whichever came first. The date of tumor relapse was determined as the day when the diagnostic examination/procedure for relapse was performed.

### Statistical analysis

Differences were compared between two groups using Kai square test or Fisher’s exact test depending on the number of each group. Cumulative survival curves were prepared using the Kaplan-Meier method and compared using the log-rank test on univariate analysis. Survival-related factors on univariate analysis (P ≤ 0.05) were entered in the multivariate Cox proportional hazards model with adjustment for age and sex. The level of significance was set at P ≤ 0.05. All statistical evaluations were performed using the SPSS 22.0 software package (SPSS Japan, Tokyo, Japan) for Windows.

## Supplementary information

Supplemental Figure 1
