# Supplementary material for: Area of residual tumor (ART) can predict prognosis after post neoadjuvant therapy resection for pancreatic ductal adenocarcinoma
Source: Sci Rep. 2019 Nov 20;9:17145. doi: 10.1038/s41598-019-53801-2 (PMC6868132; doi:10.1038/s41598-019-53801-2)
Supplement: Supplementary file 1 — Supplemental Figure 1 [file 41598_2019_53801_MOESM1_ESM.pdf]

Supplementary information

Title: Area of residual tumor (ART) can predict prognosis after post neoadjuvant therapy resection for pancreatic ductal adenocarcinoma

Author list:

Satoshi Okubo, MD,[1,2] Motohiro Kojima, MD, PhD,[1*] Yoko Matsuda, MD, PhD,[3] Kazuteru Monden, MD,[4] Yasuhiro Shimizu, MD, PhD,[5] Hirochika Toyama, MD, PhD,[6] Soichiro Morinaga, MD, PhD,[7] Naoto Gotohda, MD, PhD,[2] Katsuhiko Uesaka, MD, PhD,[8] Genichiro Ishii, MD, PhD,[1] Mari Mino-Kenudson, MD, PhD,[9] Shinichiro Takahashi, MD, PhD[2]

Supplementary figure legend:

Fig. S1. Relapse-free survival curves of post neoadjuvant resections for PDAC patients classified by ART measurement based on microscopic fields (3 40x fields)

Fig.S1

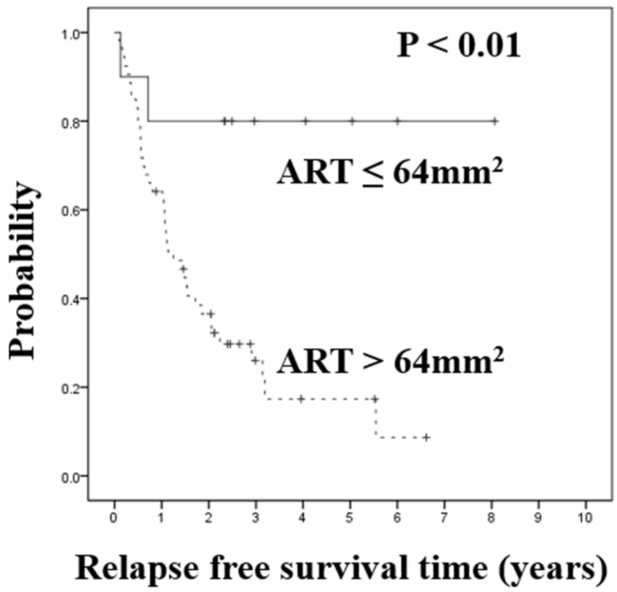
P < 0.01
ART ≤ 64mm²
ART > 64mm²
Probability
Relapse free survival time (years)
1.0
0.8
0.6
0.4
0.2
0.0
0
1
2
3
4
5
6
7
8
9
10
